# British and Foreign Review

**Published:** 1836

**Authors:** 


### Art. IV.—British and Foreign Review.

In our last number we mentioned the reception of the two first numbers of the British and Foreign Review, edited by Drs. Forbes and Conolly, of London. The able editor of the Medico Chirurgical Review characterizes the new Journal as being transcendental and exquisite—as "a splendid Bazaar of Continental commodities, of the latest fashion"—but at the same time designates it as "excellent"—and says, that far from casting an eye of envy or jealousy on this neighboring Repertorium—we welcome the stranger with the most cordial greeting."

We, not only welcome this new Journal, but warmly recommend it to our readers as a rich repertory of facts and opinions on medical subjects. The following article we have extracted from the second number of this new periodical.

*On Diseases of the Spleen, as they occur in India.* By J. O Voigt, Physician to the Danish Establishment of Frederiksnagor (Serampore.) Chronic diseases of the spleen are so frequent and fatal throughout India, especially in the low marshy provinces of Bengal, that we have good reason to complain of the neglect with which they have hitherto been treated. Most writers on the diseases of warm climates either content themselves with merely mentioning them, or else despatch them in just as unsatisfactory a manner as the authors of general systems of medicine. Even Dr. James Johnson, in his excellent work on Tropical Climates, passes them over altogether; and Amnesley's voluminous "*Researches*" and "*Sketches*," contain comparatively little on the subject. Mr. Twining, however, surgeon to the general hospital in Calcutta, has given, in the 3d vol. of the Transactions of the Medical Society of that city, a good treatise on Splenalgia.

The chief forms under which Dr. Voigt observed affections of the spleen to occur were, what he terms, *splenitis chronica*, *splenalgia congestionis*, and *lien ingens.*

Chronic splenitis displays a more or less clearly marked inflammatory diathesis, and is attended with pyrexia, considerable pain, and an uncommon degree of weakness: it chiefly attacks children of European origin, and those of rich natives, as well as persons of a more advanced age who are addicted to a stimulating diet; and appears to be principally seated in the proper membrane of the spleen. The swelling, in the first stage, is never very great, and often scarcely perceptible.

Congestion of the spleen (or splenemphraxis) shows the plain marks of an atonic diathesis, and is not at all, or but to a trifling extent, attended with febrile symptoms; it does not occasion much pain, but causes a considerable tumor, which occasionally fills the whole of the epigastric and mesogastric regions; this disease most generally occurs among the natives, and is seated in the spongy parenchyma of the organ.

Enlargement of the spleen often occurs and continues for a considerable period without pain or injury to the constitution; it does not appear to be attended with any obstruction, and is observed mostly amongst elderly individuals of the natives.

1. *Chronic splenitis* generally commences with anorexia, restlessness, sleeplessness, and a peculiar irritability of mind, which is especially remarkable in children. They gradually lose all desire for play wish to be constantly carried, hang the head, cry frequently, and manifest the greatest indifference to all that passes around them. After these precursory symptoms have continued for some days, and sometimes even two or three weeks, the essential symptoms of the disease begin gradually to appear. The face becomes pale, lead colored, or sallow, and the conjunctiva of a pale bluish color. The skin, especially of the abdomen, communicates a disagreeable sensation of heat and dryness, and the pationt falls into a state of weakness greatly disproportionate to the duration and degree of the attack. The pulse is frequent and somewhat hard, and headache, a peculiar uneasiness, slight difficulty of respiration, palpitations, and occasionally pain in the left shoulder, supervene. There is a constant feeling of tenderness and weight in the left hypochondrium, which is increased by pressure, and that often so much as to cause a loud scream. On examining the patient while lying on his back, and pressing with the fingers under the false ribs of the left side, a hard tumor is felt, which is greater or less according to circumstances, but is always of much smaller dimensions than in cases of splenalgia congestionis. The erect posture is uncomfortable to him; he likes best to lie on his left side, with the body somewhat curved. Although the tongue is moist and clean, he complains of thirst, and, after his meals, of gastrodynia, flatulence, and *ardor ventriculi.* The bowels are very irregular, but generally costive; the fæces are brown, green, or sometimes black; and the urine is pale and copious. After an uncertain period, the disease terminates either by recovery, or by passing into other diseases and causing death.

2. *Splenalgia congestionis* is the most usual of the chronic spleen diseases prevalent about Serampore. Its most remarkable precursory symptoms are lassitude, heaviness, a dislike to any kind of exertion, weakness, costiveness, and a sense of heat in the left hypochondrium. These are followed after some time by a tumor in the region of the spleen, which, though tender to the touch, is by no means so much so as in chronic splenitis. On the other hand, its bulk increases to such a degree that it generally occupies the entire of the left hypochondrium, and of the epigastrium strictly so called, and sometimes of the epigastric and mesogastric regions. The skin is dry and cold, and the cutaneous veins of the abdominal region are considerably dilated, and present a reticulated appearance. The patient experiences an indescribable lassitude and weakness, together with vertigo; the lips and gums lose their color; and the face becomes dingy, sallow, and cachectic. The bowels are tardy and constipated; the fæces dark colored; the urine pale and copious; the pulse small and feeble; the tongue generally clean and moist, and the appetite good and sometimes even voracious; but the process of assimilation goes on very imperfectly. In proportion as the strength diminishes, the flatulence, anxiety, and irritability increase; the extremities are reduced to skin and bone; cough and dyspnœa set in, and the feet become œdematous. The pulse is sometimes quickened towards evening, and the patient then suffers from an insupportable inward beat, which prevents sleep. He cannot bear to lie upon his right side, and sometimes not even on his back, but finds lying on his left side with the body curved the least uneasy posture. When walking, he generally bends to the left, and supports the left hypochondrium with his hand. The disease in females is generally attended with amenorrhœa. These symptoms, which, with a few exceptions, appear in every case of simple congestion of the spleen, continue till the disease is removed, or terminates in others, and eventually in death.

3. *Enlargement of the spleen* is generally observed amongst elderly natives, and is undoubtedly in most cases a sequela of mild intermittent fevers. The tumor is soft, not of very great magnitude, bears pressure, and produces no inconvenience except a sensation of weight. It does not appear probable that there can be any obstruction existing in such cases, which probably are of the same nature as those mentioned by Regia (Specim. observat. anat. and pathol. Ticin. 1784), in which, according to that author, the vessels can be beautifully injected.

The cachexia connected with *splenalgia Bengalensis* frequently manifests itself by a malignant ulceration, the disposition to which is so great that leechbites and blisters occasionally give rise to foul or phagedenic ulcers, which, in cases where mercury has been employed, and the patient is in a swampy situation and debarred from free access of air, occasionally terminate fatally. *Stomacace* also, both simple and scorbutic, often occurs among the poorer natives; and, although most authors recommend mercury in splenalgia, it is an indisputable fact that even a very small quantity (a few grains for instance) generally occasions a profuse salivation, and occasionally so violent a *stomacace mercurialis*, that mortification sets in, the teeth drop out, the bones become carious, and death ensues.

We have given the description of the symptoms thus at length, as well because it is the most circumstantial that we have met with, as that it may have the effect of inducing the profession in those countries to pay more attention to diseases of the spleen than they have hitherto done. From contemplating a disease in its most exquisite form, as existing where circumstances are most favorable to its development, we are the better able to judge of its pathology, and of its bearings in various respects; and it is not unlikely that, even in this country, many symptoms, generally attributed to dyspepsia, &c., may arise from a milder form of the chronic splenitis above described. We learn too that this disease may exist to a considerable extent without producing any very perceptible tumor; and on the whole it is not unreasonable to conclude that a chronic morbid action, sufficient to produce considerable uneasiness and constitutional disturbance may be going on in the spleen, for some time, without giving rise to any symptoms of such a nature as to lead us to suspect its seat to be in the organ.

As the author, on account of the prejudices of both natives and Europeans, has not had the opportunities that English medical men attached to the army or to hospitals in India have enjoyed, he is obliged to refer to them for a description of the appearances observed in the spleen after death. Proceeding next to the etiology of the disease, he contradicts Annesley's assertion respecting its comparative infrequency in India, and states that, at least in Bengal, the case is quite the reverse, both according to his own experience and that of Mr. Twining. The predisposing causes he mentions are: 1, season; splenalgia, though occurring at all times of the year, being most prevalent towards the conclusion of the periodic rain, and at the commencement of the cold season: 2, age and sex; children and delicate young men being most subject to it, while elderly persons, and women after puberty, are comparatively exempt: 3, low swampy situations, overgrown with jungle: 4, perhaps sol-lunar influence.

The most usual occasional causes are: 1, tedious remittent and intermittent fevers: 2, want of exercise and proper clothing, unwholesome diet, and intemperance in eating; these causes often produce an idiopathic splenalgia, among the natives: 3, abuse of stimulants, especially arrack: 4, opium-eating: 5, every thing that has a tendency to debilitate the system in general, and the digestive system in particular, such, for instance, as depressing passion, &c.: 6, external injuries, such as contusions from falls; but this cause does not often come into play.

In speaking of the proximate cause of splenalgia, there is a remarkable coincidence in opinion between the author and Dr. Hodgkin, in his Essay on the Uses of the Spleen. After stating that it has been proved by the experiments of Defermon on animals, that the spleen is capable of admitting a considerable variation in its size, and that its vessels can bear to be greatly dilated for some time without producing any local or general obstruction, he proceeds to observe that it is evident that, under particular circumstances, in the cold stage of intermittent fever for instance, there is a large quantity of blood thrown in upon the abdominal viscera, which would have a most destructive effect upon the more important organs, if the spleen, from its spongy and elastic texture, were not adapted to serve as a temporary reservoir for a considerable portion of the superabundant fluid: and that repeated and prolonged congestions of this description must, in persons who have been exposed to miasmata and other debilitating causes, produce a state of irritation of the organ which may terminate in chronic inflammation, or in an incurable induration.

*Treatment.* The disease is always difficult to cure, mostly very obstinate, and frequently fatal. Accordingly Dr. V. describes, at a length commensurate with the difficulty and importance of the subject, the treatment that has been found most successful in its different forms; and makes some useful practical remarks on the inconveniences attending other modes that have been proposed by different individuals: the use of mercury, for instance, he most strongly reprobates. The measures he recommends, besides attention to the diet, &c., are to attack the inflammatory symptoms, when such are present, by leeches, gentle purgatives, tepid baths, and the application to the region of the spleen of a cloth dipped in nitro-muriatic acid considerably diluted with water, and covered with an emollient cataplasm; both to be frequently changed.

The febrile irritation and local inflammation once removed, the next indication is to endeavor to diminish the bulk of the spleen. This is most likely to be effected by the continued use of purgatives combined with tonics, and by certain topical applications. He considers the preparations of iron in general, and the sulphate more especially, to have some specific influence on the spleen; but advises caution in its application, as an overdose readily produces cardialgia, gastrodynia, vomiting, and diarrhœa; and it is absolutely contra-indicated where there is any febrile irritation present, as well as in hepatic, pleuritic, and dysenteric complications. The other internal remedies are aloes and myrrh, combined with senna, gentian, carminatives, diuretics, and diaphoretics; nitric acid; and tartar emetic in small doses. The topical applications are, friction with the flesh-brush; nitro-muriatic epithems; small blisters; stimulating liniments to the abdomen; and moxas.

The treatment of chronic splenitis or of congestion of the spleen, difficult enough when they are simple, is rendered still more so by their complication with other diseases. For instance, in *splenalgia hepatica*, mercury, though indicated for the liver, cannot be employed, as it increases the spleen disease.

In another part of the paper Dr. Voigt makes the following remarks on the terminations of splenalgia. "In Bengal, it rarely runs on to suppuration, but, when not fatal, it generally terminates in induration, if not cured in time. The febrile symptoms then disappear, and the pain in the left hypochondrium is much diminished, but the tumor remains and becomes hard and distinct. The health improves, and, with the exception of costiveness, a sensation of fulness and weight under the left false ribs, a dry cough, some dyspnœa, and occasionally a slight pain shooting to the scapula, the patient feels pretty well, and may live on for many years in that condition. He is however generally predisposed by it to fever, liver complaints, dysentery, dropsy, and cholera, by some one or other of which he is at last carrried off. Splenalgia, especially when complicated, sometimes runs its course very rapidly, and may terminate in death in three weeks or a month. Œdema of the feet and legs, ascites, dysentery, echymoses, a malignant stomacace, and singultus, are the general precursors of death in subacute cases, whilst obstinate diarrhœa and hectic close the scene in the most usual form, splenalgia congestionis. Persons who have once had chronic disease of the spleen are very liable to be attacked by it again.—*Bibliothek for Læger, No.* 2, 1834. Copenhagen.

